# An audience research study to disseminate evidence about comprehensive state mental health parity legislation to US State policymakers: protocol

**DOI:** 10.1186/s13012-017-0613-9

**Published:** 2017-06-26

**Authors:** Jonathan Purtle, Félice Lê-Scherban, Paul Shattuck, Enola K. Proctor, Ross C. Brownson

**Affiliations:** 1 Department of Health Management and Policy, Dornsife School of Public Health, Drexel University, 3215 Market St, Philadelphia, PA 19104 USA; 2 Department of Epidemiology and Biostatistics, Dornsife School of Public Health, Drexel University, 3215 Market St, Philadelphia, PA 19104 USA; 3 A.J. Drexel Autism Institute, Drexel University, 3215 Market St, Philadelphia, PA 19104 USA; 4 Center for Mental Health Services Research, Brown School of Social Work, Washington University in St. Louis, St. Louis, MO USA; 5 Prevention Research Center in St. Louis, Brown School of Social Work, Washington University in St. Louis, St. Louis, MO USA; 6 Division of Public Health Sciences and Alvin J. Siteman Cancer Center, Washington University School of Medicine, Washington University in St. Louis, St. Louis, MO USA

**Keywords:** Dissemination, Policy dissemination research, Audience research, Parity legislation, US State policymakers

## Abstract

**Background:**

A large proportion of the US population has limited access to mental health treatments because insurance providers limit the utilization of mental health services in ways that are more restrictive than for physical health services. Comprehensive state mental health parity legislation (C-SMHPL) is an evidence-based policy intervention that enhances mental health insurance coverage and improves access to care. Implementation of C-SMHPL, however, is limited. State policymakers have the exclusive authority to implement C-SMHPL, but sparse guidance exists to inform the design of strategies to disseminate evidence about C-SMHPL, and more broadly, evidence-based treatments and mental illness, to this audience. The aims of this exploratory audience research study are to (1) characterize US State policymakers’ knowledge and attitudes about C-SMHPL and identify individual- and state-level attributes associated with support for C-SMHPL; and (2) integrate quantitative and qualitative data to develop a conceptual framework to disseminate evidence about C-SMHPL, evidence-based treatments, and mental illness to US State policymakers.

**Methods:**

The study uses a multi-level (policymaker, state), mixed method (QUAN→qual) approach and is guided by Kingdon’s Multiple Streams Framework, adapted to incorporate constructs from Aarons’ Model of Evidence-Based Implementation in Public Sectors. A multi-modal survey (telephone, post-mail, e-mail) of 600 US State policymakers (500 legislative, 100 administrative) will be conducted and responses will be linked to state-level variables. The survey will span domains such as support for C-SMHPL, knowledge and attitudes about C-SMHPL and evidence-based treatments, mental illness stigma, and research dissemination preferences. State-level variables will measure factors associated with C-SMHPL implementation, such as economic climate and political environment. Multi-level regression will determine the relative strength of individual- and state-level variables on policymaker support for C-SMHPL. Informed by survey results, semi-structured interviews will be conducted with approximately 50 US State policymakers to elaborate upon quantitative findings. Then, using a systematic process, quantitative and qualitative data will be integrated and a US State policymaker-focused C-SMHPL dissemination framework will be developed.

**Discussion:**

Study results will provide the foundation for hypothesis-driven, experimental studies testing the effects of different dissemination strategies on state policymakers’ support for, and implementation of, evidence-based mental health policy interventions.

**Electronic supplementary material:**

The online version of this article (doi:10.1186/s13012-017-0613-9) contains supplementary material, which is available to authorized users.

## Background

Inadequate insurance coverage for mental health services is a major barrier to accessing mental health care in the USA [1–7]. A significant coverage-related barrier to care is insurance providers’ practice of imposing restrictions on the utilization of mental health services that are more restrictive than those for physical health services (e.g., fewer covered mental health visits, higher deductibles and copays for mental health services) [8, 9]. Federal legislation has attempted to curb this practice [10, 11], but substantive discrepancies in coverage of mental health versus physical health services persist [12–14]. As a result, a substantial portion of the 19% of the US population with a mental illness [15] has restricted access to mental health services, including evidence-based treatments (EBTs), and the benefits they produce.

Comprehensive state mental health parity legislation (C-SMHPL) is an evidence-based policy intervention that enhances mental health insurance coverage and improves access to mental health services, and thus also EBTs [16]. C-SMHPL is defined as public policy that requires insurers to provide the same level of coverage for all mental health and physical health benefits with no discrepancy [17]. C-SMHPL is recommended by the US Community Preventive Services Task Force [18] an independent, non-government panel of experts that provides evidence-based recommendations about preventive interventions. Systematic reviews demonstrate that C-SMHPL increases coverage and the utilization of mental health services and does not significantly increase insurance premium costs [16, 19]. Despite strong evidence of C-SHMPL’s benefits, only 19 states have implemented C-SMHPL as of December 2015 [17].

US State policymakers have the exclusive authority to implement C-SMHPL. Research on physical activity [20, 21] and cancer [22–24] policy interventions has found that policymaker support is integral to implementation of evidence-based policies and that support can be cultivated through dissemination strategies that target knowledge and attitudes, and account for state-level contexts [25]. Thus, C-SMHPL uptake might be improved by dissemination strategies that target state policymakers’ knowledge deficiencies and attitudinal barriers and are targeted to state-level factors related to C-SMHPL support.

Surveys of health care providers and the general public have found low-levels of knowledge about C-SMHPL and that support varies according to attitudes about mental illness [26–29], but no data exist on state policymakers’ knowledge or attitudes about C-SMHPL or how these attributes are associated with support. More broadly, reviews [30–34] highlight that few studies have focused on the dissemination of mental health evidence to policymakers, in the USA or abroad. Furthermore, US State policymakers are an understudied audience in the field of dissemination research [25, 34] and minimal guidance exists to inform the design of strategies to disseminate evidence about C-SMHPL, and more generally information about mental health and EBTs, to this audience [35].

**Fig. 1:**
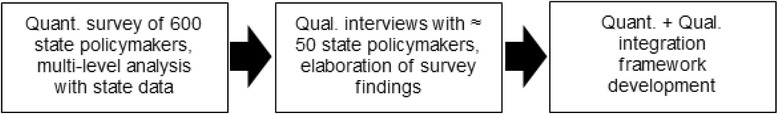
Shows the squence of study methods. Quant. = Quantitative, Qual. = Qualitative

The current study aims to address these knowledge gaps through an exploratory audience research study that uses a multi-level (policymaker, state), sequential mixed method (QUAN→ qual) design with the ultimate goal of developing an empirically grounded framework to guide the dissemination of evidence about C-SMHPL, EBTs, and mental illness to US State policymakers (Fig. 1).

### Mental health parity laws in the USA

For nearly 50 years, federal and state legislation in the USA has attempted to promote equal insurance coverage (i.e., “parity”) of mental and physical health benefits [36]. Parity coverage has been a focus of three federal laws: the 1996 Mental Health Parity Act, the 2008 Mental Health Parity and Addiction Equity Act, and the 2010 Patient Protection and Affordable Care Act. Although these laws have increased coverage for mental health services, they have been limited in their ability to achieve equal coverage because most authority to regulate insurance exists at the state-level. C-SMHPL is needed to supplement federal parity laws, help achieve equal coverage, and therein maximize access to mental health services and EBTs. Table 1 defines the three major types of state parity laws and shows their implementation status across the USA.

**Table 1:** Types of state mental health parity legislation and implementation status in the USA, December 2015

Type	Definition	States implemented
C-SMHPL	Health insurance providers are required to provide the same level of coverage for all mental and physical health benefits (e.g., identical deductibles, copayments, visit limits, and lifetime/ annual limits) with no discrepancy	AL, AR, CT, DE, HI, ID, IL, MD, MN, NJ, NC, OH, OK, RI, SD, VT, VA, WV, WY
Limited-SMHPL	Health insurance providers are required to provide the same level of coverage for some mental and physical health benefits (e.g., identical deductibles, copayments, visit limits, and lifetime/ annual limits) with some discrepancy	AK, CA, IN, IA, KS, ME, MA, MI, MS, MO, MT, NE, NV, NH, ND, OR, PA, TN, TX, WI
Non-parity	Health insurance providers have the option to provide the same level of coverage for some MH and physical health benefits (e.g., identical deductibles, copayments, visit limits, and lifetime/annual limits) with full discrepancy	AZ, CO, FL, GA, KY, LA, NM, NY, SC, UT, WA

*C-SMHPL* comprehensive state mental health parity legislation

Source: National Conference of State Legislatures [17]

### Evidence base for comprehensive state mental health parity legislation

In 2012, the US Community Preventive Services Task Force classified C-SMHPL as an evidence-based intervention and recommended its widespread implementation [16]. The recommendation was based on a review of 30 studies which found strong evidence that C-SMHPL expands insurance coverage, increases utilization of mental health services, and improves financial protections for people with mental illness [18]. For example, the review found that C-SMHPL increased the proportion of employed adults with coverage for mental health services by a median of 13.6 percentage points. An economic review concluded that C-SMHPL does not substantially increase insurance provider or beneficiary costs [19]. C-SMHPL also has potential to de-institutionalize the stigma of mental illness [37–39].

### Factors related to comprehensive state mental health parity legislation implementation

#### Individual-level knowledge and attitudes among the public and policymakers

Although no studies have investigated knowledge or attitudes related to C-SMHPL among state policymakers, studies of non-policymaker provide indication of factors that might be associated with C-SMHPL support. The US public has limited knowledge about parity laws and support varies according to attitudes about mental illness. Only 7% of adults have heard of “mental health parity” and, more broadly, the US public has limited knowledge about mental illness and EBTs [28, 40–42]. A 2013 survey found that 69% of US adults supported the concept of parity and that higher levels of mental illness stigma, defined as negative attitudes towards people with mental illness [39], were associated with less support [26]. This finding is consistent with evidence indicating that mental illness stigma is highly prevalent and associated with lower levels of support for mental health interventions [37–39]. Studies show that conservative political ideology is associated with higher mental illness stigma while personal experience with mental illness is associated with lower levels of stigma and higher support for C-SMHPL [28, 43, 44]. Focusing events, such as mass shootings, increase public concern about mental illness and support for policies to address it [45–49], although these policies are often not evidence-based [50, 51]. Understanding if and how these factors are associated with state policymakers’ support for C-SMHPL can contribute to the development of targeted, effective dissemination strategies.

#### State-level social, economic, and political contexts

One study investigated state-level factors associated with C-SMHPL implementation [52]. Hernandez and Uggen analyzed longitudinal ecologic data and found that high state unemployment and conservative political leadership reduced the likelihood of C-SMHPL implementation. Although the study did not collect data from actual state policymakers, it provides indication of contextual factors that might influence state policymakers decisions related to C-SMHPL.

### Audience research to enhance policymaker-focused dissemination strategies

C-SMHPL has tremendous potential to increase access to EBTs and improve population mental health. In order for C-SMHPL to be brought to scale; however, information about it must be effectively disseminated to US State policymakers. Dissemination does not occur spontaneously [53] and the ‘passive’ dissemination strategies typically used by researchers (e.g., scientific publications, conference presentations) are generally ineffective at reaching policymakers and satisfying their information needs [54, 55]. Contextual factors such as political climate, economic environment, and public opinion often further complicate the transmission of research evidence and implementation of evidence-based policies [25, 56–59]. Targeted dissemination strategies can bridge the gap between the production of mental health evidence and implementation of evidence-based mental health policies.

Audience research is a critical first step in developing these strategies [60–62]. Audience research is defined as the formative assessment of an audience’s knowledge and attitudes about an issue or preferences for receiving information about it [62]. In accordance with the National Institutes of Health, dissemination research is defined as the study of how, why, and under what circumstances information on evidence-based interventions spreads. As an audience research dissemination study, the current study will characterize US State policymakers’ knowledge and attitudes about C-SMHPL and identify individual- and state-level attributes associated with support for C-SMHPL. Study findings will inform the design and testing of strategies to disseminate evidence about C-SMHPL, EBTs, and mental illness to US State policymakers.

### Specific aims

#### Aim 1

Characterize state policymakers’ knowledge and attitudes about C-SMHPL and identify individual- and state-level attributes associated with support for C-SMHPL.

#### Rationale for Aim 1

State policymaker support for C-SMHPL is essential to its widespread implementation. Knowledge and attitudes are mutable constructs that influence support and can be modified through targeted dissemination strategies. Yet no data exist to inform the design of C-SMHPL dissemination strategies for state policymaker audiences.

#### Aim 2

Integrate quantitative and qualitative data to develop a conceptual framework to disseminate C-SMHPL evidence to state policymakers.

#### Rationale for Aim 2

A framework is needed to effectively disseminate evidence to policymakers. Frameworks exist to guide the dissemination of information about physical health interventions to policymakers [21], but none are focused on information about mental health interventions, such as C-SMHPL. A targeted dissemination framework is needed because policymakers likely have stigmatizing attitudes towards people with mental illness [37–39, 43, 63–65] and limited knowledge about mental health research evidence [28, 40–42].

### Contribution to dissemination science

By achieving these aims, the current study addresses important knowledge gaps and advances dissemination science in at least four ways. First, policymakers are essential to maximizing the scale-up of EBTs, but are an understudied audience in dissemination and implementation research—especially in the USA [34]. A review of projects funded through the NIH’s Dissemination and Implementation Research in Health Program Announcements between 2007 and 2014 found that only 12 (8.2%) of the projects were focused on public policy, just four (3.3%) of which were classified as dissemination research [34]. Policymaker-focused dissemination research is a more developed field in Australia [66, 67] and Canada [68] but it is not clear whether or how results from these studies are applicable to US State contexts.

Furthermore, policymaker-focused dissemination research conducted in the USA and abroad has largely been limited to physical, not mental, health issues. A systematic review of interventions aimed at increasing the use of research evidence in mental health policymaking did not identify any studies that collected data from US policymakers [30], results consistent with two earlier reviews [32, 69] The lack of mental health-focused dissemination research with US State policymakers is compounded by an absence of frameworks to guide dissemination to this audience. A review of 61 dissemination and implementation frameworks found few that addressed dissemination at the policy-level and none that were focused on mental health evidence [33]. As a result, sparse empiric guidance exists to inform strategies to disseminate evidence on C-SMHPL and mental health to US State policymakers. The current study will address these knowledge gaps by collecting and integrating primary data (quantitative and qualitative) from US State policymakers.

Second, the current study will conduct audience research to enhance the reach and spread of C-SMHPL—an evidence-based intervention that has not been previously studied in dissemination research. C-SMHPL has been the focus of numerous outcome evaluations [16], but no studies have investigated how knowledge, attitudes, and other contextual factors are associated with C-SMHPL support among state policymakers—the audience with exclusive implementation authority. By focusing on C-SMHPL and policymakers, the current study will support the scale-up of C-SMHPL and ultimately the reach of EBTs.

Third, the current study is the first to investigate US State policymakers’ knowledge and attitudes about mental health, EBTs, and mental illness stigma. These topics have been extensively studied among clinicians [70–73] and the general public [26, 40, 43, 46, 74], but not state policymakers. Although the current study is specifically focused on C-SMHPL, it will produce an empirically grounded framework that can help guide the dissemination of a wider range of mental health research findings to state policymakers.

Fourth, the current study employs a multi-level design that is infrequently used in policy dissemination research. The few dissemination studies conducted with US State policymakers have been limited to a focus on individual-level factors. The current study nests policymakers’ survey and interview responses within ecologic measures of the state contexts in which they reside and make policy decisions related to C-SMHPL and EBTs. The multi-level design allows for identification of how individual- and state-level factors jointly influence support for C-SMHPL and the design and testing of targeted dissemination strategies that account for contextual factors at the state-level.

## Methods

### Practice partners

The current study was conceptualized in collaboration with five practice partner organizations that will serve in an advisory capacity throughout the project. The partner organizations are the National Conference of State Legislatures (NCSL), a non-governmental organization that serves as an informational resource for US State legislators and their staff; the National Association of State Mental Health Program Directors, a member organization that represents the executives of state public mental health service systems; the National Association of Insurance Commissioners, an organization created and governed by states’ chief insurance regulators that provides guidance on best practices for the regulatory oversight of insurance markets; Mental Health America, a national advocacy organization focused on addressing the needs of people affected by mental illness; and Parity Track, an organization that works to monitor and elevate implementation of federal and state parity laws.

### Conceptual framework

**Fig. 2:**
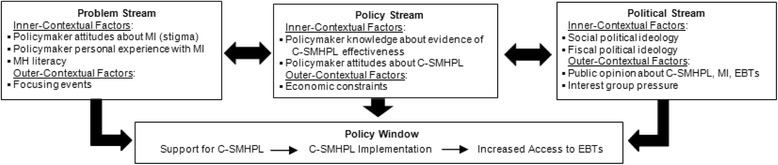
MH = mental health, MI = mental illness, EBTs = evidence-based mental health treatments, C-SMHPL = comprehensive state mental health parity legislation. * Adapted from Kingdon’s Multiple Streams Framework of the policymaking process [75], adapted to incorporate constructs from Aarons’ Model of Evidence-Based Implementation in Public Sectors [71]

Few dissemination frameworks are focused on US State policymakers [33] and none are specifically designed to disseminate information about evidence-based mental health policy interventions (e.g., C-SMHPL) to this audience. Thus, the current study is guided by Kingdon’s Multiple Streams Framework [75] and adapted to incorporate constructs from Aarons’ Model of Evidence-Based Implementation in Public Sectors [71] (Fig. 2). Multiple Streams is a political science framework founded on the premise that countless issues are constantly competing for policymakers’ attention. When policy, problem, and political “streams” converge around an issue a “policy window” opens and policy is implemented to address it. In the current study, the *problem* is inadequate insurance coverage for mental health services, the *policy* is C-SMHPL, and the *politics* are public opinion and interest group pressure related to C-SMHPL, mental illness, and EBTs.

Multiple Streams is regarded as a dissemination framework [33], but was not created with the intent of structuring dissemination research [53]. For the purpose of the current study, a deficit of Multiple Streams is that it does not account for individual attributes of state policymakers (i.e., the adopters of C-SMHPL). These characteristics are important because they are mutable targets that can be altered by targeted dissemination strategies [28]. Thus, Multiple Streams was supplemented with the concepts of inner- and outer-contextual factors from Aarons’ Model of Evidence-Based Implementation in Public Sectors [71]. Inner-contextual factors are individual attributes of the adopters of an intervention, whereas outer-contextual factors constitute features of the external environment that affect adoption. Figure 2 depicts inner- and outer-contextual factors within each of the three streams postulated, on the basis of extant research [26–29, 37–43, 45–49, 52, 63–65, 70–74], to be associated with state policymaker support for C-SMHPL. Supplementing Multiple Streams is acceptable because the framework is rated as having high construct flexibility for dissemination research [33].

### Aim 1 methods

Aim 1 methods consist of a quantitative survey of 600 US State policymakers—500 state legislators (i.e., elected policymakers) and 100 administrators (i.e., appointed policymakers)—collection of state-level ecologic data, and exploratory multi-level regression analyses to identify inner- and outer-contextual factors associated with policymaker support for C-SMHPL.

#### Policymaker survey instrument and measures

Table 2 shows policymaker-level (i.e., inner-contextual) variables, how they will be measured, and the sources from which items are derived (legislator survey as Additional file 1). The primary dependent variable is global support for C-SMHPL and the secondary dependent variables are support for specific benefits covered at parity (e.g., co-pays, visit limits) and mental health conditions and substance use disorders covered at parity (e.g., major depression disorder, opioid use disorder). A definition of C-SMHPL will be provided towards the beginning of each survey.

**Table 2:** Inner-contextual policymaker-level variables

Domain	Sub-domain	# Items	Measure	Item sources^a^
*Dependent variables*				
C-SMHPL	Global support for C-SMHPL	1	5-point Likert	[26, 28]
	Support for specific MH/SUD benefits covered at parity	5	5-point Likert	
	Support for specific MH/SUD conditions covered at parity	6	5-point Likert	
*Independent variables*				
C-SMHPL	Awareness of C-SMHPL	1	Dichotomous	[17, 18, 28, 29]
	Knowledge about C-SMHPL parity research evidence	2	5-point Likert	
MH/SUD literacy	Knowledge about effectiveness of MH/SUD treatments	2	5-point Likert	[41, 42, 74, 99]
	Knowledge about prevalence of MI/SUDs among US adults	2	7 nominal, select 1	
	Knowledge about impact of child trauma on MI/SUD risk	4	5-point Likert	
	Knowledge of ACE study	1	Dichotomous	
Mental illness stigma	Mental illness stigma	4	5-point Likert	[26]
Personnel experience with MH/SUD treatments	Known someone who sought treatment for MH/SUD issue	2	Dichotomous	[64]
	Personally sought treatment for MH/SUD issue	2	Dichotomous	
Research dissemination preferences	Importance of features of disseminated MH/SUD research	9	5-point Likert	[55, 100]
	Preferred/trusted sources of MH/SUD research	1	9 nominal, select 1	
MH/SUD research utilization	Frequency of MH/SUD research utilization	1	6-point Likert	[101]
	Barriers to MH/SUD research utilization	1	11 nominal, select 3	
	Influence of MH/SUD research on state policymaking	1	5 nominal, select 2	
Prioritization of MH/SUD issues	Health policy priories	1	19 nominal, select 3	[77, 78]
	Ever introduced bill on MH/SUD issue	2	Dichotomous	
Demographics (collected via survey)	Social ideology	1	7-point Likert	[55, 77, 78, 100]
	Fiscal ideology	1	7-point Likert	
	Highest level of education	1	Ordinal	
	Number of years as state legislator	1	Ordinal	
	Health committee member	1	Dichotomous	
	Insurance committee member	1	Dichotomous	
	Motivation for completing survey	1	5 nominal, select 2	
Demographics (collected via NCSL dataset)	Political party membership	1	Nominal	
	Legislative chamber	1	Dichotomous	
	Gender	1	Dichotomous	
	State	1	Nominal	

*MH* mental health, *MI* mental illness, *EBTs* evidence-based mental health treatments, *C-SMHPL* comprehensive state mental health parity legislation, *ACE* Adverse childhood experience, *US* United States, *SUD* substance use disorder, *NCSL* National Conference of State Legislatures

^a^Items adapted from these sources

The primary independent variables are knowledge and attitudes (i.e., inner-contextual factors) that are postulated to be associated with C-SMHPL support. Knowledge of C-SMHPL research evidence (i.e., that C-SMHPL increases insurance coverage for mental health services and does not increase premium costs) and mental health literacy (e.g., knowledge about the prevalence of mental illness and attitudes about the effectiveness of EBTs) will be assessed. Attitudes about people with mental illness (i.e., stigma) will be measured in addition to history of personal experience with mental health and substance use disorder treatments. Information of research dissemination preferences (e.g., important features of disseminated research, preferred sources of disseminated research) and research utilization practices will be collected in addition measures of political ideology and demographics.

#### Cognitive pre-testing of policymaker survey instrument

Many of the survey items related to C-SMHPL, mental health, and mental illness have been validated in prior research with the general public, but not policymakers. Thus, to ensure data quality, the survey instrument will be cognitively pre-tested with at least 15 state policymakers (legislative and administrative) before fielding. These policymakers will be identified and recruited in collaboration with the study’s partner organizations. In accordance with recommendations for cognitive pre-testing [76], the process will entail telephone-based interviews in which each survey item will be presented, a response will be obtained, and then questions will be asked about the item. The questions will span four domains: comprehension (e.g., what do respondents think questions are asking?), retrieval (e.g., what information do respondents need to recall to answer questions?), judgment (how do respondents formulate answers to questions?), and response (e.g., how do response options and social desirability influence the answers?). The survey instrument will be revised to address issues identified through pre-testing.

#### Survey sample selection, recruitment, and data collection

Sample frames will be constructed of the two types of state policymakers with ability to affect C-SMHPL implementation: state legislators and state administrative policymakers.

##### State legislators

There are 7383 state legislators in the USA. A list of all US State legislators as of January 2017 and their contact information will be purchased from NCSL. Newly elected state legislators serving their first term in 2017 (*n* = 1229) will be excluded from the sample frame because of their limited time in office when data collection occurs. To ensure variation in the current status of parity legislation and other state-level contextual factors that could affect survey responses, 30 state legislators will be randomly selected from each state and recruited to complete the survey (30 legislators per state * 50 states = 1500 state legislators in original sample frame). Assuming a response rate of approximately 30%, consistent with prior survey research with US State legislators [77–80], this will result in approximately 500 state legislators completing the survey. If the response rate is inadequate to achieve a sample size of 500, the sample frame will be extended and additional state legislators will be recruited.

State legislator survey data will be collected by a professional survey research firm using an assertive recruitment strategy that combines e-mail, post-mail, and telephone-based modes of recruitment and data collection. First, each legislator in the sample frame will receive a personalized post-mail and e-mail invitation describing the study and inviting them to complete the survey online using a unique ID and password. Then, over the course of the next 28 weeks, each legislator will receive two post-mailed paper versions of the survey with self-addressed postage-paid return envelopes, 10 e-mail invitations to complete the survey online, and up to 15 telephone calls in the last 4 weeks of the data collection period. In total, a legislator who does not complete the survey nor opt-out of the sample frame will be contacted a total of 29 times.

Two additional strategies will be used to increase the chances of a high response rate. First, the survey concludes with a question asking the legislator to indicate the two primary reasons for completing the survey (e.g., concern about mental health and substance abuse issues among their constituents, a desire to have research findings more effectively disseminated to them). These responses will be analyzed while the survey is still in the field and results about the motivations for survey completion will inform the messaging of recruitment materials. Second, because the dates of the legislative session vary between states, in-session or out-of-session contact information for each legislator will be used according to whether their legislature is in-session or out-of-session on the date of each contact attempt.

##### State administrative policymakers

Through discussions with practice partners, it was determined state health insurance commissioners and state mental health programs directors were the two types of state administrative policymakers with the most influence on C-SMHPL implementation decisions, through enforcement of parity laws or by serving in an advisory capacity to the state legislature. The names and contact information for these policymakers is publically available and centralized on the National Association of State Mental Health Program Directors and National Association of Insurance Commissioners websites. These two policymakers from each state (two administrative policymakers per state * 50 states = 100 state policymakers in sample frame) will be recruited by e-mail and telephone over a 29-week period to complete a web-based version of the survey. The administrative policymaker survey will be modeled after the legislator survey, but modified in collaboration with practice partners to reflect differences in the scope of authority and responsibly between the different types of policymakers.

#### State-level measures

Table 3 shows state-level (i.e., outer-contextual) variables that will be measured for all 50 states using the most recent data available. The first four variables listed were used in Hernandez and Uggen’s study of C-SMHPL implementation and will be measured using identical methods [52]. State mental health parity status (i.e., C-SMHPL, limited-SMHPL, non-parity) will be obtained from NCSL [17]. Interest group pressure for mental health parity will be measured by the number of years that the National Alliance on Mental Illness (NAMI) has been registered each state [81]. Economic pressure against mental health parity will be measured by seasonally adjusted state unemployment rates for the month prior to when each survey is completed [82]. State government ideology will be measured using a composite index based on state legislative voting [83] and state government partisanship will be measured according to NCSL’s designation based on controlling political party in control of the state’s legislature and governor’s office [84].

**Table 3:** Outer-contextual state-level variables

Domain	Measure	Data source
*Independent variables*		
State MH parity status^a^	Nominal: C-SMHPL; limited-SMHPL; non-parity law in place in respondent’s on date of survey completion	National Conference of State Legislatures [17]
Interest group pressure for C-SMHPL^a^	Continuous: Number of years that the National Alliance on Mental Illness has been in existence in respondent’s state on date of survey completion	Encyclopedia of Associations: Regional, State and Local Organizations [81]
Economic pressure against C-SMHPL^a^	Continuous: Seasonally adjusted unemployment rate in respondent’s state in most recent full month prior to the date of survey completion	US Bureau of Labor Statistics [82]
State government ideology^a^	Continuous: Government ideology in respondent’s state as measured by roll-call voting scores of state congressional delegations (range 1 to 100) in most recent full year prior to the date of survey completion	Berry et al. 2010 [83]
State government partisanship	Nominal: Republican control (Republicans hold majority of seats in state legislature, Republic Governor); Democrat control (Democrat hold majority of seats in state legislature, Democrat Governor); Divided control (one party holds majority of seats in state legislature, Governor is of a different party) in respondent’s state on date of survey completion	National Conference of State Legislatures [84]
State prioritization of access to MH services	Ordinal: Quartile rank of composite Access to Care Score (based on nine metrics) for respondent’s state for most recent year available prior to the date of survey completion	Mental Health America [87]
Mass shootings as focusing events that affect attitudes towards MH and MI	Continuous: Number of mass shooting^b^ events in respondent’s state in the two years prior to the date of survey completion Continuous: Number of people injured mass shooting events in respondent’s state in the two years prior to the date of survey completion	Stanford Mass Shootings of America Database [86]

MH = mental health, MI = mental illness, EBTs = evidence-based mental health treatments, C-SMHPL = comprehensive state mental health parity legislation

^a^Used in Hernandez and Uggen’s study of C-SMHPL implementation [52]

^b^A mass shooting is defined as an event in which three or more people are injured

Given the increased incidence of mass shootings in the USA [85] and research indicating these events generate public support for policies to address mental illness [46–49], the Stanford Mass Shootings of America Database [86] will be used to measure (a) the number of mass shooting events and (b) the number of people injured in these events, within each state in the 2 years prior to the date when the survey is completed. A mass shooting is operationalized in the database as an event in which three or more people were injured. Seventy-seven of these events occurred in 2014 and 2015, suggesting adequate variability between states. Lastly, state prioritization of mental health will be measured using the quartile rank of each state’s Mental Health America Access to Care Score, a composite score based on nine metrics [87].

#### Quantitative analysis

A single de-identified dataset will be created in which survey responses from each policymaker will be linked to data on the state-level variables for the state in which they reside. The legislator survey includes 59 items and nine a priori domains. Cronbach’s alpha will be used to statistically confirm whether survey items assess a similar construct. First, variables will be characterized using descriptive statistics (e.g., frequencies, central tendencies) and plots (e.g., stem and leaf plots, q-q plots). Then, multi-level regression models will be used to investigate the relative strength of policymaker- versus state-level measures in determining support for C-SMHPL. The models will also produce estimates and standard errors accounting for the hierarchical nature of the data. Multi-level multinomial and binary logistic regression models will be used for each of the three dependent variables (i.e., global support for C-SMHPL, support for specific benefits covered at parity, support for specific conditions covered at parity) to estimate odds ratios (ORs) of each level of support compared to strong opposition.

For each outcome, the intra-class correlation coefficient will first be calculated to estimate the proportion of variability attributable to policymaker- versus state-level variation. Adjusted multi-level random effects regression models will then be used to estimate ORs for associations of the policymaker- and state-level measures with the outcome. A sequential model-building approach will be used in which blocks of policymaker-level variables will first be entered according to domain (e.g., mental illness stigma) followed by the addition of state-level variables.

##### Statistical power

The total sample of 600 US State policymakers will provide sufficient power to detect anticipated effect sizes of policymaker and state-level measures. Alpha at 0.05 and prevalence of strong support for C-SMHPL at 50% will provide 88% power to detect a 40% reduction in odds of support associated with a standard deviation difference in a state-level variable. Power analyses were conducted using Optimal Design Plus Empirical Evidence Software, Version 3.01 [88].

### Aim 2 methods

Aim 2 methods consist of semi-structured interviews with approximately 50 US State policymakers to develop a framework to disseminate evidence about C-SMHPL, EBTs, and mental illness to this audience. All Aim 2 methods will be informed by quantitative findings from Aim 1 and begin after preliminary quantitative analyses are complete.

#### Interview guide development

A semi-structured interview guide will be developed in collaboration with practice partners to shed additional light on quantitative findings (sample interview questions in Additional file 2). The guide will contain seven to ni ne primary open-ended questions, with multiple follow-up and probing questions, and span domains such as experiences with C-SMHPL, perceptions of barriers and facilitators to C-SMHPL implementation, opinions about EBTs and mental illness, and processes through which research influences (and does not influence) state mental health policymaking.

#### Interview respondent selection, recruitment, and data collection

The interview sample frame will consist of all policymakers who completed a quantitative survey (Aim 1). A purposive sampling strategy will be used in which respondents will be selected based on known characteristics, not at random [89]. In accordance with recommendations for mixed method research [90, 91], policymakers will be selected based on characteristics that will allow us to explore findings that emerge from quantitative analyses.

Interviews will be conducted until “saturation” is achieved (i.e., the point at which no new information is yielded from the data) [92]. Research shows that meaningful themes within sub-groups typically emerge after six interviews and saturation often occurs after 12 interviews [92]. Recruiting from the two policymaker populations (i.e., legislative and administrative) and allowing for three sub-group comparisons within each population, between 36 and 72 interviews [2 × 3 × (6 to 12) = 36 to 72] will be conducted in total. Each respondent will be contacted ten times in a 3-week period before selecting an alternate. Interviews will be approximately 30 min in duration, telephone-based, audio recorded, transcribed verbatim, and imported into NVivo 10, a qualitative data management program.

#### Qualitative analysis and framework development

Qualitative analysis will be structured around the objective of developing a framework to disseminate evidence about C-SMHPL, EBTs, and mental illness to US State policymakers and guided by Jabareen’s procedure for conceptual framework development [93]. Accordingly, qualitative analysis will progress over the course of six steps.

- Step 1: Review quantitative findings and existing dissemination frameworks. This step serves to identify, a priori, concepts that have potential utility in the framework. Key findings from quantitative analyses will be transformed into preliminary concepts with names and definitions (e.g., mental illness stigma/C-SMHPL support association). Using previous reviews [33, 94], existing dissemination frameworks will be reviewed to identify key dissemination concepts (e.g., message source). This step will orient activities in Steps 2 through 4.
- Step 2: Read, code, and categorize interview data. This step consists of organizing interview data into categories at a low-level of abstraction. All interview transcripts will be read by two coders who will assign sections of text to inductively generated categories in NVivo [91]. Coders will meet regularly to discuss coding decisions and establish category names and definitions. Coders will write memos throughout the coding process to capture ideas (e.g., variations in themes by state-level context) and discuss these memos with the project team to refine categories [95].
- Step 3: Establish core concepts. This step entails coding transcripts at a higher level of abstraction and creating core concepts that reflect commonalities between multiple categories [96]. Concepts will be created through an iterative process using analytic techniques such as coding matrixes, quote tables, and querying for divergent findings [95]. After core concepts have been named and defined, two coders will re-read and code all transcripts and kappa statistics of inter-rater reliability will be calculated for each concept. Concepts with less than “moderate agreement” (kappa ≤ .60) [97] will be discarded.
- Step 4: Create framework. The purpose of this step is to synthesize quantitative and qualitative findings and create a conceptual framework that provides a comprehensive understanding of how evidence about C-SMHPL, EBTs, and mental illness can be most effectively disseminated to US State policymakers. To achieve this, diagrams will be created that depict sequences, contingencies, and inter-relationships among concepts at and between policymaker- and state-levels.
- Step 5: Validate framework. This step uses “member checking,” a qualitative validation technique in which research participants review and comment on the study’s findings [98], to assess whether the framework makes sense to policymakers. To elicit feedback and while accommodating policymakers busy schedules, an asynchronous strategy will be used in which the framework diagram and accompanying narrative will be e-mailed to interview respondents with an accompanying request for them to provide feedback via brief, open-ended questions that will be answered through a web-based survey. Responses will be imported into the NVivo database and analyzed using thematic content analysis.
- Step 6: Revise framework. At this step, in collaboration with practice partners, feedback obtained in Step 5 will be integrated and the framework will be revised accordingly.

### Summary of activities to mix quantitative and qualitative data and methods

To reiterate, quantitative and qualitative data and methods will be mixed in three ways. First, quantitative findings will inform qualitative sampling by providing policymaker- and state-level criteria for selecting interview respondents and aiding the identification policymakers who meet these criteria. Second, quantitative findings will inform qualitative data collection as qualitative interview questions will be developed to elaborate upon quantitative findings. Third, quantitative findings will be transformed into qualitative concepts that will be used in the qualitative conceptual framework development process.

## Discussion

If successful, the current project will advance the US National Institute of Mental Health’s (NIMH) Objective to “Strengthen the Public Health Impact of NIMH-Supported Research” by producing knowledge that will enhance the dissemination of C-SMHPL evidence to state policymakers and therefore scale-up a policy intervention that expands access to mental health services and EBTs. Study results will provide the foundation for hypothesis-driven, experimental studies testing the effects of different dissemination strategies on state policymakers’ knowledge and attitudes about, support for, and implementation of, evidence-based mental health policy interventions.

There is at least one aspect of the current study that warrants additional consideration—the national political context at the time of data collection and emergent demands on US State legislators that could potentially contribute to a sub-optimal response rate. Survey data will be collected in spring and summer 2017, a time of elevated political turmoil and policy change in the USA. A new President and broad proposed changes in the federal administration and its policies (e.g., health insurance reform, environmental regulation, immigration enforcement, state funding) are likely to place increased pressure on state legislators to respond to constituents needs and develop new legislation. By straining their already finite time resources, these demands could potentially make state legislators less likely to complete the survey. The study’s assertive (i.e., up to 29 contact attempts) and multi-modal (i.e., telephone, post-mail, e-mail) recruitment strategy should maximize the chances of obtaining a satisfactory response rate despite these challenges.

## Additional files

Additional file 1: Legislator survey instrument. (DOCX 53 kb)

Additional file 2: Sample interview questions. (DOCX 49 kb)

## Abbreviations

C-SMHPL: Comprehensive state mental health parity legislation
EBTs: Evidence-based mental health treatments
NIH: National Institute of Health
NIMH: National Institute of Mental Health
