# Supplementary material for: An audience research study to disseminate evidence about comprehensive state mental health parity legislation to US State policymakers: protocol
Source: Implement Sci. 2017 Jun 26;12:81. doi: 10.1186/s13012-017-0613-9 (PMC5485547; doi:10.1186/s13012-017-0613-9)
Supplement: Supplementary file 2 — Sample interview questions. (DOCX 49 kb) [file 13012_2017_613_MOESM2_ESM.docx]

**SUPPLEMENTAL File 2: sample Interview Questions**

**Experiences with C-SMHPL:**

- As you might know, C-SMHPL was recently passed in your state. Tell me about the process through which C-SMHPL came to become law.
  - How did research evidence about C-SMHPL influence this process?
  - What organizations disseminated research evidence about C-SMHPL?
  - Did people provide oral testimony during legislative hearings about C-SMHPL? If so, who provided it and did it influence your decision making? Why or why not?
- Tell me what you know about C-SMHPL.
  - How did you learn this information?

**Perceptions of barriers and facilitators to C-SMHPL implementation:**

- Your state was an early adopter of C-SMHPL. What factors do you think contributed to this?
- Your state does not have any mental health parity law. Why do you think that this is the case?
- What would be needed for state agencies to effectively enforce C-SMHPL if it were to become law in your state?

**Opinions about mental illness and evidence-based mental health treatments:**

- In your opinion, what makes a mental health treatment “evidence-based?”
- To what extent do you think that evidence-based mental health treatments can help people to recover from mental illnesses?
  - What factors do you think contribute to whether or not mental health treatments help people to recover?
- To what extent do you think that early interventions can prevent mental illnesses?
  - What factors do you think contribute to whether or not early interventions can prevent mental illnesses

**Processes through which research influences mental health policymaking:**

- What are some characteristics of research that make it useful in mental health policymaking processes?
  - Information about the cost implications of research findings?
  - Research is conducted by researchers at a university in your state?
  - Research conducted on a population in your state?
- What are some things that researchers can do to more effectively communicate mental health research to policymakers?
